# Convergent and divergent patterns of morphological differentiation provide more evidence for reproductive character displacement in a wood cricket *Gryllus fultoni *(Orthoptera: Gryllidae)

**DOI:** 10.1186/1471-2148-9-27

**Published:** 2009-02-01

**Authors:** Yikweon Jang, Yong-Jin Won, Jae Chun Choe

**Affiliations:** 1 Department of Life Sciences and Division of EcoScience, Ewha University, Seoul, 120-750, Republic of Korea

## Abstract

**Background:**

In ecological character displacement, traits involved in reproductive isolation may not evolve in arbitrary directions when changes in these traits are by-products of adaptation to an ecological niche. In reproductive character displacement, however, selection acts directly on reproductive characters to enhance the degree of reproductive isolation between sympatric populations. Thus, the direction of change in reproductive characters may be arbitrary in relation to changes in other morphological characters. We characterized both tegminal characters and characters indicative of body size in sympatric and allopatric populations of *Gryllus fultoni*, a species displaying character displacement in its calling song characters in areas of sympatry with *G. vernalis *populations, to infer the nature and direction of selection acting on reproductive and morphological characters in sympatry.

**Results:**

Except for mirror area, the number of teeth in a file, and ovipositor length of *G. fultoni*, all male and female morphological characters in *G. fultoni *and *G. vernalis *exhibited a uniform tendency to decrease in size with increasing latitude. There was no significant variation in female morphological characters between sympatric and allopatric *G. fultoni *populations. However, males of sympatric and allopatric *G. fultoni *populations significantly differed in head width, hind femur length, and mirror area even after controlling for clinal factors. Head width and hind femur length of *G. fultoni *were more similar to those of *G. vernalis *in sympatric populations than in allopatric populations, resulting in morphological convergence of *G. fultoni *and *G. vernalis *in sympatry. However, the mirror area of *G. fultoni *displayed the divergent pattern in relation to the sympatric *G. vernalis *populations.

**Conclusion:**

Divergence-enhancing selection may be acting on mirror area as well as calling song characters, whereas local adaptation or clinal effects may explain variation in other morphological characters in sympatric populations of *G. fultoni*. This study also suggests that structures and behaviors that directly enhance reproductive isolation may evolve together, independently of other morphological traits.

## Background

Once the subject of controversy, character displacement, a pattern in which the difference between two species is accentuated in areas of sympatry and is reduced in areas of allopatry [1,2], is now recognized as a powerful force driving trait diversification and even speciation in sympatry [3-8]. The divergence of characters is driven by selection against interspecific resource competition in ecological character displacement (ECD) or by selection against costly hybridization in reproductive character displacement (RCD). In the case of ECD, reproductive isolation evolves as a by-product of adaptation to different ecological niches [9,10]. In Darwin's finches, for example, the size and shape of the beak reflect adaptation to the nature of food exploited by each species [11]. Body size or morphological characters indicative of body size are often critical determinants of resource use [12-18]. The diversification of beak morphology and body size has shaped patterns of vocal signal evolution, resulting in reproductive isolation and speciation in Darwin's finches [19].

In the case of RCD, reproductive isolation is achieved by direct selection on reproductive characters in areas of sympatry such that the reproductive characters shift away from the reproductive characters of the sympatric species as well as those of allopatric populations of the same species. Reproductive traits that are directly targeted by selection to enhance reproductive isolation in sympatry may be correlated with other morphological traits such as body size, as seen in ECD. For example, in insect and frog species whose communication modality for mate attraction is mainly acoustic signals, there is a strong correlation between body size and carrier frequency [20]. In general, the larger the animals are, the lower their carrier frequencies are. Accordingly, if there is pressure to alter the carrier frequency, body size may change in accordance with the change in the carrier frequency. However, the direction of change in reproductive characters may also be arbitrary relative to changes in other morphological characters between populations in sympatry when the nature of selection is different in reproductive and other morphological characters or when there is selection only on reproductive characters.

Unfortunately, demonstration of RCD to date has mostly involved only the identification of patterns of variation in characters of interest between areas of sympatry and allopatry. Most studies of RCD have considered morphological or behavioral characters directly related to reproduction only, disregarding other morphological characters that may also be potentially important for reproductive isolation (but see [18]). This narrow focus may be prevalent because documented cases of RCD largely involve behavioral traits, or because it is difficult to determine which morphological traits may be associated with RCD. The latter issue is particularly problematic for researchers studying mechanisms of evolution of reproductive isolation between populations in sympatry [21].

**Figure 1:**
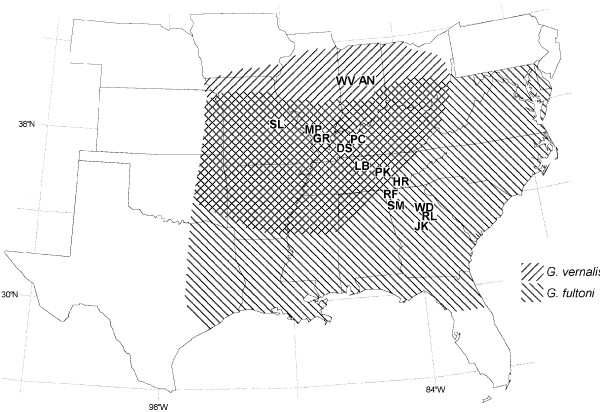
**Geographic distributions of *Gryllus fultoni *and *G. vernalis*, which occur in the eastern United States**. The right- and left-hatched areas denote distributions of *G. fultoni *and *G. vernalis*, respectively. The cross-hatched area represents the sympatric zone. Crickets of far allopatric *G. fultoni *were collected in JK, RL, and WD. Crickets of near allopatric *G. fultoni *were collected in SM, RF, and HR. Sympatric localities of *G. fultoni *and *G. vernalis *were LB, PK, DS, PC, GR, MP, and SL. Crickets of allopatric *G. vernalis *were collected in AN and WV. See the main text for the abbreviations of localities.

In the eastern United States, two cricket species, *Gryllus vernalis *Blatchley and *G. fultoni *(Alexander) (Orthoptera: Gryllidae), occur together in an area between eastern Kansas and the Appalachian Mountains (Fig. 1; [22,23]). *G. fultoni *and *G. vernalis *range south and north, respectively, from the sympatric zone. The two species have very similar calling song structures consisting of three-pulse chirps. An examination of geographic variation in calling songs revealed that the distributions of two calling song characters, chirp rate and pulse rate, showed a pattern consistent with RCD in *G. fultoni *[23,24]. That is, there was little or no overlap in these two characters in sympatric populations of these two species, but these two characters overlapped extensively in allopatric populations of *G. fultoni *and sympatric populations of *G. vernalis *[23]. A detailed analysis revealed that *near allopatric *populations, allopatric populations located close to the sympatric area, had chirp and pulse rates whose values were intermediate between those of sympatric and *far allopatric *populations, allopatric populations located relatively far from the area of sympatry. The divergence of pulse and chirp rates in sympatry seems to be under genetic control, based on a common-environment rearing study [23]. Results of playback experiments showed that *G. fultoni *females in sympatric and near allopatric populations did not orient to heterospecific stimuli, which should significantly reduce heterospecific mating attempts in sympatry, consistent with the predictions of RCD [24]. A population genetic study using the mitochondrial cytochrome *c *oxidase subunit I gene showed that all haplotypes of *G. fultoni *from sympatric populations were separated from those of allopatric populations, with sympatric populations forming a distinct clade (S.-I. Lee, unpublished data). Field and laboratory recordings showed no differences in calling song characters between sympatric and allopatric populations of *G. vernalis *[25]. However, *G. vernalis *females discriminated against heterospecific males in close-range mating behaviors.

Here we measured morphological characters of *G. fultoni *and *G. vernalis *in areas of sympatry and allopatry (Table 1) to understand the patterns of variation of these characters given the divergent pattern of pulse and chirp rates in sympatric and allopatric *G. fultoni *populations. A divergent pattern occurs when the characters of closely related species are more dissimilar in areas of sympatry than areas of allopatry, whereas in a convergent pattern, the characters are more similar in areas of sympatry than in areas of allopatry. Morphological characters that we measured can be classified as tegminal characters, those that may be responsible for calling song production, and non-tegminal characters, which may be related to overall body size in crickets [26]. Patterns of variation in reproductive and morphological characters may be used to infer the nature and direction of selection acting on these characters in sympatry.

**Table 1:** Collecting localities of *G. fultoni *and *G. vernalis*.

Species	patry	Locality	*n*_fm_	*n*_ff_	*n*_vm_	*n*_vf_	City, State
*G. fultoni*	far allopatry	JK	23	18			Jackson, Georgia
		RL	12	19			Rutledge, Georgia
		WD	0	11			Winder, Georgia
	
	near allopatry	SM	11	8			Summerville, Georgia
		RF	19	19			Rising Fawn, Georgia
		HR	18	15			Harrison, Tennessee

*G. fultoni *and *G. vernalis*	sympatry	LB	12	0	4	4	Lebanon, Tennessee
		DS	18	10	7	17	Dawson Springs, Kentucky
		PC	7	0	0	0	Park City, Kentucky
		GR	21	12	8	18	Goreville, Illinois
		MP	5	5	12	10	Murphysboro, Illinois
		SL	18	3	0	1	Sullivan, Missouri
		PK	0	0	6	14	Pikeville, Tennessee

*G. vernalis*	allopatry	AN			12	11	Anderson, Indiana
		WV			14	16	Waveland, Indiana

*n*_fm_, *n*_ff_, *n*_vm_, and *n*_vf _indicate the numbers of *G. fultoni *males, *G. fultoni *females, *G. vernalis *males, and *G. vernalis *females used for morphological measurements, respectively. Populations of *G. fultoni *were grouped into three zones: far allopatric, near allopatric, and sympatric zone [23]. Populations of *G. vernalis *were grouped into two zones: allopatric and sympatric zone [25].

## Results

### Male Morphological Characters of *G. fultoni*

Mean values and standard deviations for morphological characters are shown in Table S1 (additional file 1). Results of the multivariate GLM revealed that the latitude was a significant factor for head width in *G. fultoni *(Table 2). Elevation was a significant factor for head width and thorax length. Head width, thorax length, hind femur length, and mirror area were significantly different among the *G. fultoni *populations. Pairwise comparisons of male morphological characters showed significant differences between far allopatric and sympatric populations in head width, hind femur length, and mirror area (Table 3). There were also significant differences between far allopatric and near allopatric populations in head width, thorax length, hind femur length, and mirror area (Table 3). In *G. vernalis*, latitude and elevation were significant factors for both head width and mirror area. In addition, elevation was a significant factor for harp area. Zone had a significant effect for head width and mirror area in *G. vernalis *(Table 2).

**Table 2:** The analyses of the multivariate general liner models on male morphological variables.

		*G. fultoni*		*G. vernalis*	
			
Source	Variable	*df*	Mean Square *F P*	*df*	Mean Square *F P*
Zone	Head Width	2	0.442 4.440 **0.013**	1	0.552 6.179 **0.016**
	Thorax Length	2	0.787 3.850 **0.023**	1	0.137 1.012 0.319
	Hind Femur Length	2	2.717 4.475 **0.013**	1	0.948 1.309 0.257
	Harp Area	2	1.113 0.769 0.465	1	0.792 1.428 0.237
	Mirror Area	2	1.197 3.244 **0.042**	1	1.863 9.359 **0.003**
	Number of Teeth in a File	2	65.797 0.793 0.454	1	286.720 1.920 0.171

Latitude	Head Width	1	0.666 6.688 **0.011**	1	0.437 4.887 **0.031**
	Thorax Length	1	0.556 2.721 0.101	1	0.046 0.341 0.562
	Hind Femur Length	1	2.166 3.568 0.061	1	0.159 0.220 0.641
	Harp Area	1	2.191 1.515 0.220	1	0.691 1.246 0.269
	Mirror Area	1	0.546 1.481 0.225	1	1.704 8.558 **0.005**
	Number of Teeth in a File	1	142.861 1.748 0.188	1	410.985 2.753 0.102

Longitude	Head Width	1	0.033 0.333 0.565	1	0.024 0.269 0.606
	Thorax Length	1	0.148 0.724 0.396	1	0.050 0.370 0.546
	Hind Femur Length	1	0.523 0.862 0.355	1	0.365 0.504 0.480
	Harp Area	1	0.069 0.048 0.827	1	0.470 0.848 0.361
	Mirror Area	1	< 0.001 < 0.001 0.998	1	0.011 0.055 0.816
	Number of Teeth in a File	1	4.597 0.056 0.813	1	327.734 2.195 0.144

Elevation	Head Width	1	1.609 16.146 **< 0.001**	1	0.451 5.053 **0.028**
	Thorax Length	1	4.762 23.300 **< 0.001**	1	0.085 0.006 0.937
	Hind Femur Length	1	1.431 2.357 0.127	1	0.339 0.468 0.497
	Harp Area	1	2.304 1.593 0.209	1	4.192 7.558 **0.008**
	Mirror Area	1	1.074 2.913 0.090	1	1.915 9.618 **0.003**
	Number of Teeth in a File	1	151.037 1.848 0.176	1	3.610 0.024 0.877

Error	Head Width	158	0.100	58	0.089
	Thorax Length	158	0.204	58	0.135
	Hind Femur Length	158	0.607	58	0.724
	Harp Area	158	1.446	58	0.555
	Mirror Area	158	0.369	58	0.199
	Number of Teeth in a File	158	81.724	58	149.305

The analyses were conducted separately for *G. fultoni *(*n *= 164) and *G. vernalis *(*n *= 63). The predictor variable was zone, and covariates were latitude, longitude, and elevation. Numbers in bold indicate significance at the level of 0.05.

**Table 3:** Post hoc analyses of pairwise comparisons of male morphological characters in *G. fultoni*.

	far allopatric vs.	near allopatric vs.	far allopatric vs.
	near allopatric	sympatric	Sympatric
Head Width	0.380 ± 0.128, **0.003**	0.226 ± 0.151, 0.136	0.606 ± 0.237, **0.012**
Thorax Length	0.447 ± 0.183, **0.016**	0.019 ± 0.216, 0.929	0.428 ± 0.339, 0.209
Hind Femur Length	0.789 ± 0.315, **0.013**	0.961 ± 0.372, **0.011**	1.750 ± 0.585, **0.003**
Harp Area	0.587 ± 0.487, 0.230	0.458 ± 0.574, 0.426	1.045 ± 0.903, 0.249
Mirror Area	0.623 ± 0.246, **0.012**	0.397 ± 0.290, 0.173	1.020 ± 0.456, **0.027**
Number of Teeth in a File	4.584 ± 3.659, 0.212	1.915 ± 4.318, 0.658	6.499 ± 6.787, 0.340

The analyses were based on the multivariate general linear model (*n *= 164; see Tables 2). Populations of *G. fultoni *were grouped into three zones: far allopatric, near allopatric, and sympatric zones. Each cell contains the mean differences of two groups |first row – second row| ± standard error and its significance level. Numbers in bold indicate significance at the level of 0.05.

**Figure 2:**
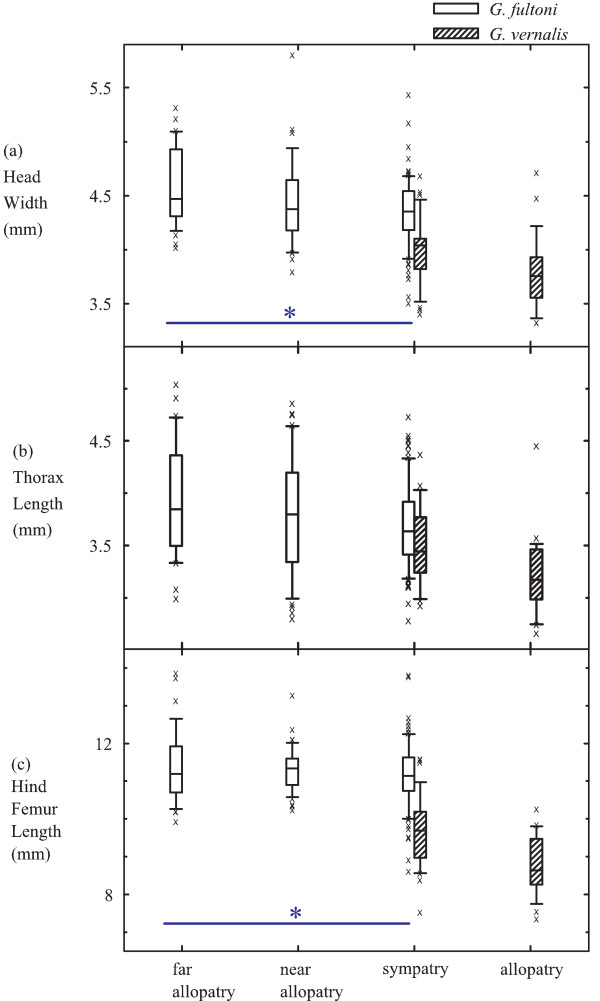
**Distributions of male morphological characters that were indicative of body size in *G. fultoni *(open) and *G. vernalis *(hatched)**. Box plots show distributions of head width (a), thorax length (b), and hind femur length (c). Top, middle, and bottom lines of the boxes indicate 75 percentile, median, and 25 percentile, respectively. The upper and lower whiskers indicate 90 and 10 percentiles, respectively. X denotes an outlier. Localities of far allopatric *G. fultoni *populations were JK, RL, and WD, and localities of near allopatric *G. fultoni *populations were SM, RF, and HR. Sympatric localities of *G. fultoni *and *G. vernlais *were LB, DS, PC, GR, MP, SL, and PK. Localities of allopatric *G. vernalis *localities were AN and WV. See the main text for the abbreviations of localities. * indicates that there was a significant difference for the character among *G. fultoni *populations.

**Figure 3:**
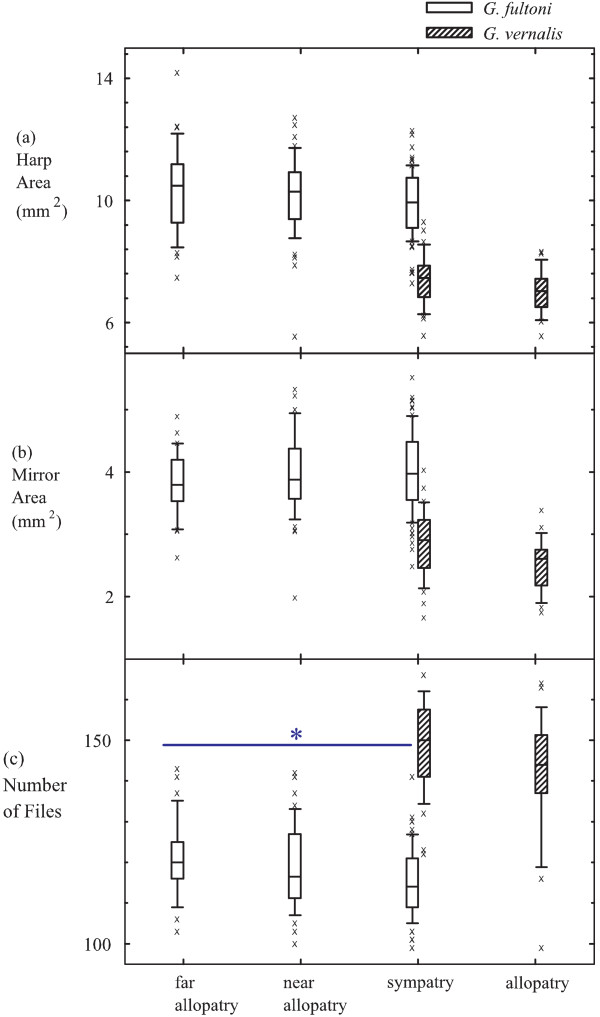
**Distributions of male tegminal characters in *G. fultoni *(open) and *G. vernalis *(hatched)**. Box plots show distributions of harp area (a), mirror area (b), and the number of teeth in a file (c). Top, middle, and bottom lines of the boxes indicate 75 percentile, median, and 25 percentile, respectively. The upper and lower whiskers indicate 90 and 10 percentiles, respectively. X denotes an outlier. Localities of far allopatric *G. fultoni *populations were JK, RL, and WD, and localities of near allopatric *G. fultoni *populations were SM, RF, and HR. Sympatric localities of *G. fultoni *and *G. vernlais *were LB, DS, PC, GR, MP, SL, and PK. Localities of allopatric *G. vernalis *localities were AN and WV. See the main text for the abbreviations of localities. * indicates that there was a significant difference for the character among *G. fultoni *populations.

There was a convergent pattern in head width, thorax length, hind femur length, and harp area between *G. fultoni *and *G. vernalis *populations (Fig. 2a, 2b, 2c, and Fig. 3a). That is, the smallest difference in these characters occurred between sympatric *G. fultoni *and *G. vernalis *populations, whereas the greatest difference was between allopatric populations (additional file 1, Table S1). By contrast, the number of teeth in a file diverged in sympatry for *G. fultoni *and *G. vernalis *populations (Fig. 3c). That is, the smallest difference in the number of teeth in a file occurred between far allopatric *G. fultoni *and allopatric *G. vernalis *populations, whereas the greatest difference occurred between sympatric *G. fultoni *and *G. vernalis *populations (additional file 1, Table S1). However, there was no statistical difference in this character among *G. fultoni *populations (Table 2). Mirror area, which was statistically different between far allopatric and sympatric *G. fultoni *populations, followed neither convergent nor divergent patterns as defined in the Background (Fig. 3b). However, the difference in average values for mirror area was greatest between sympatric *G. fultoni *and sympatric *G. vernalis *populations and was smallest between far allopatric *G. fultoni *and sympatric *G. vernalis *populations. Thus, the distribution of mirror areas among *G. fultoni *populations effectively followed a divergent pattern in relation to those of the sympatric *G. vernalis *populations. Therefore, the pattern of variation in mirror area, which may be functionally related to production of calling songs, was similar to the patterns of variation in pulse and chirp rate between allopatric and sympatric populations of *G. fultoni*, which is consistent with RCD. However, all non-tegminal characters both in *G. fultoni *and *G. vernalis *showed a uniform tendency of decrease with increasing latitudes, suggesting clinal effects on these characters.

### Female Morphological Characters

Mean values and standard deviations for female morphological characters are shown in Table S1 (additional file 1). In *G. fultoni*, latitude was a significant factor for thorax length, and longitude was a significant factor for ovipositor length. Other than that, there was no significant predictor variable or covariates for female morphological characters (Table 4). In *G. vernalis*, latitude and longitude were significant factors for head width. Zone had no significant effect for all female morphological characters of *G. fultoni*, but zone had a significant effect for head width in *G. vernalis *(Table 4). All morphological characters except for ovipositor length showed a uniform tendency to decrease with increasing latitude, suggesting clinal effects on these characters both in *G. fultoni *and *G. vernalis*. The ovipositor length in *G. fultoni *seemed to show a divergent pattern, but there was no statistical difference between sympatric and allopatric populations.

**Table 4:** The analyses of the multivariate general liner models on female morphological variables.

		*G. fultoni*		*G. vernalis*	
			
Source	Variable	*df*	Mean Square *F P*	*df*	Mean Square *F P*
Zone	Head Width	2	0.127 0.737 0.481	1	1.823 10.140 **0.002**
	Thorax Length	2	0.323 1.609 0.205	1	0.097 0.599 0.441
	Hind Femur Length	2	1.265 1.312 0.273	1	1.580 1.631 0.205
	Ovipositor Length	2	0.973 0.311 0.733	1	1.589 1.739 0.191

Latitude	Head Width	1	0.478 2.782 0.098	1	1.437 7.992 **0.006**
	Thorax Length	1	1.227 6.115 **0.015**	1	0.009 0.054 0.816
	Hind Femur Length	1	0.008 0.009 0.925	1	0.428 0.442 0.508
	Ovipositor Length	1	2.549 0.816 0.368	1	1.182 1.293 0.259

Longitude	Head Width	1	0.206 1.196 0.276	1	0.804 4.472 **0.037**
	Thorax Length	1	0.017 0.085 0.771	1	0.020 0.125 0.725
	Hind Femur Length	1	0.096 0.100 0.753	1	0.483 0.498 0.482
	Ovipositor Length	1	13.142 4.205 **0.043**	1	0.675 0.739 0.393

Elevation	Head Width	1	0.018 0.103 0.749	1	0.080 0.445 0.507
	Thorax Length	1	0.004 0.022 0.883	1	0.083 0.510 0.477
	Hind Femur Length	1	0.064 0.067 0.797	1	0.631 0.651 0.422
	Ovipositor Length	1	0.253 0.081 0.777	1	0.040 0.044 0.834

Error	Head Width	114	0.172	86	0.180
	Thorax Length	114	0.201	86	0.162
	Hind Femur Length	114	0.964	86	0.968
	Ovipositor Length	114	3.126	86	0.914

The analyses were conducted separately for *G. fultoni *(*n *= 120) and *G. vernalis *(*n *= 91). The response variables included head width, thorax length, hind femur length, and ovipositor length. The predictor variable was zone, and covariates were latitude, longitude, and elevation. Numbers in bold indicate significance at the level of 0.05.

## Discussion

The most obvious pattern of morphological variation in both *G. fultoni *and *G. vernalis *is latitudinal variation in body size. Values of all characters other than mirror area, number of teeth in a file, and ovipositor length in *G. fultoni *decreased with increasing latitude. In the northern hemisphere, the length of the season favorable for development and reproduction generally decreases with increasing latitude. A reduction in season length in turn generally corresponds to a decrease in body size [27-31]. Because of this latitudinal variation in non-tegminal characters, *G. fultoni *and *G. vernalis *were more similar in body size in areas of sympatry than in areas of allopatry. Thus, this convergent pattern in non-tegminal characters may simply reflect local responses to an environmental gradient [32]. However, natural selection might also have promoted convergence in the non-tegminal characters in areas of sympatry for the two cricket species as a result of greater ecological similarity in these areas [33]. Morphological similarity may also have resulted from introgressive hybridization between the two species in areas of sympatry [34,35]. If substantial introgression of some genes but not others occurred [36-40], this could result in dissimilarity in sound-producing structures and behaviors but similarity in other morphological traits between *G. fultoni *and *G. vernalis *in areas of sympatry.

Among the three characters that showed significant differences among *G. fultoni *populations, the pattern of variation in the mirror area was in the opposite direction from the general tendency for morphological variation in *G. fultoni *and *G. vernalis*. However, the distribution of mirror areas did not strictly follow a divergent pattern, which was defined as displaying more dissimilarity in sympatry than in allopatry for both taxa. Nonetheless, we believe that the mirror area of *G. fultoni *has been under selection to diverge in sympatry, because the greatest difference in mirror area was observed in the sympatric populations of *G. fultoni *and *G. vernalis*. It is appropriate to compare variation in mirror area among *G. fultoni *populations to sympatric *G. vernalis *populations, because sympatric populations of the two species are most likely to interact with each other.

The divergent pattern in mirror area suggests that a force distinct from clinal variation or local adaptation may be operating on this trait. Previous field and laboratory studies of calling songs revealed that pulse and chirp rates of *G. fultoni *also diverged from those of sympatric *G. vernalis *populations in areas of sympatry, a pattern consistent with RCD [23]. Thus, there were similar patterns of divergence in both calling song characters and the morphological features that may be responsible for the production of calling songs, which suggests that the same selection pressures may affect both calling song characters and mirror area in the sympatric populations of *G. fultoni*. Playback experiments also revealed that female preferences shifted in accordance with changes in male calling song characters in the sympatric *G. fultoni *populations [24]. As we controlled for clinal factors in our analyses of geographic variation in morphological characters and calling song characters [23], these pressures may result from selection against costly interspecific mating between *G. fultoni *and *G. vernalis *in sympatry [41-43]. At present, however, premating reproductive isolation between these two species appears to be complete [23]. Thus, if selection against interspecific mating played a significant role in producing the current pattern of differentiation in calling song characters and mirror area in *G. fultoni*, the process probably largely resulted from selection in the past, rather than the present.

Alternatively, shifts in mirror area and calling song characters in sympatric populations of *G. fultoni *may have originated from competition for resources [1,2]. Such resource competition causes divergence in feeding morphology in sympatric populations of *G. fultoni*; these morphological changes may also affect calling song characteristics, leading to increased reproductive isolation [9,44,45]. However, more detailed studies of feeding morphology in *G. fultoni *and *G. vernalis *will be necessary to understand the role, if any, of ECD in promoting divergence in calling song characters and mirror area in areas of sympatry.

While three male non-tegminal characters showed variation among far allopatric, near allopatric, and sympatric *G. fultoni *populations, female morphological characters did not differ among these populations. One possible explanation for this absence of variation is that selection on females favors large body size, which often covaries with number and size of eggs that they can produce [27,46,47]. If female crickets can maximize their fitness by maintaining the largest body size possible, then their body sizes might not show much geographic variation.

Our findings suggest that characters that may enhance reproductive isolation may evolve independently of other characters in areas of sympatry. In this study, selection against heterospecific mating seemed to favor divergence in calling songs, female preferences for calling songs, and morphological characters responsible for the production of calling songs in *G. fultoni *in areas of sympatry with *G. vernalis*, whereas selection pressures related to clinal variables may have been more important for variation in other male morphological characters of *G. fultoni *and all morphological characters of *G. vernalis*. Furthermore, comparisons of morphological characters suggest that different selection pressures may have acted on *G. fultoni *males and females across areas of sympatry and allopatry.

## Conclusion

The pattern of morphological differentiation in sympatry and allopatry adds one more line of evidence for reproductive character displacement in the acoustic communication of *G. fultoni*. Mirror area, which may be responsible for production of calling songs, showed a divergent pattern in areas of sympatry that was consistent with the patterns of variation in two calling song characters and patterns of female mate preference in *G. fultoni*. However, local adaptation or clinal effects may explain variation in other morphological characters in sympatric populations of *G. fultoni*. Furthermore, this study suggests that traits that may enhance reproductive isolation may evolve independently from other morphological traits in areas of sympatry.

## Methods

### Study Species

*G. fultoni *and *G. vernalis *occur in forests and adjacent fields of eastern North America (Fig. 1; [22,23]). *G. fultoni *and *G. vernalis *have similar life histories and morphologies. Both species overwinter as juveniles and are generally univoltine [48]. In addition, both species are strictly micropterous in the field and have a narrower head than pronotum. Genetic studies suggest that while *G. fultoni *and *G. vernalis *occur in the same clade, they are not sister taxa ([25,49]; D. Gray, personal communication).

Field collection, rearing, and maintenance of crickets were described in Jang and Gerhardt [23,24,50] and Jang et el. [25]. Crickets described as "far allopatric" *G. fultoni *were collected in three localities: Jackson, Georgia (JK); Rutledge, Georgia (RL); Winder, Georgia (WD). The letter in parentheses represents the abbreviation of the locality. Crickets described as "near allopatric" *G. fultoni *were collected in three localities: Summerville, Georgia (SM); Rising Fawn, Georgia (RF); Harrison, Tennessee (HR). Crickets of both *G. fultoni *and *G. vernalis *described as "sympatric" were collected in six localities: Lebanon, Tennessee (LB); Pikeville, Tennessee (PK); Dawson Springs, Kentucky (DS); Park City, Kentucky (PC); Goreville, Illinois (GR); Murphysboro, Illinois (MP); Sullivan, Missouri (SL). Crickets of allopatric *G. vernalis *were collected in two localities: Anderson, Indiana (AD); Waveland, Indiana (WV).

### Morphological Measurements

All morphological measurements were conducted on the first-generation offspring of the field-caught females. We measured the following male morphological characters: head width, thorax length, hind femur length, harp area, mirror area, and the number of teeth in a file. In female crickets, we measured head width, thorax length, hind femur length, and ovipositor length. Each cricket was placed in the solution of 75% ethanol. The head was removed from the thorax and placed with compound eyes facing a stereoscopic zoom microscope (Nikon Inc. model SMZ800; Tokyo, Japan) lens. Digital images of the morphological characters were then captured with a color CCD camera (ARTRAY Co. Ltd. model: ARTCAM-150P; Tokyo, Japan) and output to a PC computer. We measured the size of each morphological feature except ovipositor length from the images using ART Image (version 2; Gendis Co., Ltd.; Seoul, Korea). Head width was defined as the distance between the outer edges of the compound eyes. Thorax length was the distance between the anterior and posterior ends in the midline of the thorax. A right hind leg was removed from the thorax and placed on its side. Hind femur length was measured from the base of the femur to the joint with the tibia. If a right hind leg was not present, a left hind leg was used for measurement instead. Ovipositor length was measured as the distance between the beginning of the external section of the ovipositor from the point of emergence from the abdomen and the tip of the ovipositor. Ovipositor length was measured with a digital venire caliper.

In crickets the file is the Cu2 vein on a tegmen that consists of a series of teeth and is struck by the plectrum of the opposite tegmen to produce sound pulses [51]. The harp is a triangular area enclosed by the Cu1 and Cu2 veins on the male tegmina. The mirror is an elliptical area, which is located at the distal end of the harp. The harp and mirror may be responsible for radiating sound produced by the file and plectrum on tegmina in crickets. Bennet-Clark [52] determined that the major elastic components of the resonant system are the file and the first anal vein and that the mass component is the combined mass of the file, anal area, and harp. However, the resonance of the mirror was not studied in detail by Bennet-Clark [52], so its importance has not yet been fully determined. To measure harp area, mirror area, and the number of teeth in a file, a right tegmen was removed. Harp and mirror areas were calculated from digital images of the right tegmen using the built-in area function on the ART Image. On the digital images of the file clearly visible teeth were counted to calculate the number of teeth in a file.

### Statistical Analysis

Because of significant correlations among morphological characters, we used multivariate general linear models (GLM) to test whether far allopatric, near allopatric, and sympatric populations of *G. fultoni *differed in morphological characters. The predictor variable for the multivariate GLM was zone, which indicated whether a population of *G. fultoni *was far allopatric, near allopatric, or sympatric. The spatial variables, latitude, longitude, and elevation (hereafter referred to as clinal variables) were covariates. For male crickets, the response variables included head width, thorax length, hind femur length, harp area, mirror area, and the number of teeth in a file. For female crickets, the response variables included head width, thorax length, hind femur length, and ovipositor length. Where significant differences among populations were detected, we conducted post hoc pairwise comparisons among far allopatric, near allopatric, and sympatric populations of *G. fultoni*.

## Authors' contributions

YJ participated in the design of the study and the writing of the manuscript, as well as conducting all cricket sampling in the field and performing all statistical analyses. Y-JW participated in the design of the study and the writing of the manuscript, as well as conducting all morphological measurements. JCC coordinated the entire project and participated in writing of the manuscript. In addition, all authors read and approved the final manuscript.

## Supplementary Material

Additional file 1 **Title: Descriptive statistics of morphological characters in *G. fultoni *and *G. vernalis*.** The values in a cell are mean ± standard deviation. See the Method for definitions of morphological characters. See Table 1 for sample sizes. Click here for file
