# Supplementary material for: Convergent and divergent patterns of morphological differentiation provide more evidence for reproductive character displacement in a wood cricket Gryllus fultoni (Orthoptera: Gryllidae)
Source: BMC Evol Biol. 2009 Feb 1;9:27. doi: 10.1186/1471-2148-9-27 (PMC2640364; doi:10.1186/1471-2148-9-27)
Supplement: Additional file 1 — Title: Descriptive statistics of morphological characters in G. fultoni and G. vernalis. The values in a cell are mean ± standard deviation. See the Method for definitions of morphological characters. See Table 1 for sample sizes. [file 1471-2148-9-27-S1.doc]

Table S1. Descriptive statistics of morphological characters in *G. fultoni* and *G. vernalis*.

|  | morphological characters | *G. fultoni* | | |  | *G. vernalis* | |
| --- | --- | --- | --- | --- | --- | --- | --- |
|  |  | far allopatric | near allopatric | sympatric |  | sympatric | allopatric |
| male | Head Width | 4.5577 ± 0.3490 | 4.4434 ± 0.3725 | 4.3434 ± 0.3190 |  | 3.9902 ± 0.2973 | 3.7699 ± 0.3245 |
|  | Thorax Length | 3.9207 ± 0.5267 | 3.8021 ± 0.5654 | 3.6897 ± 0.4074 |  | 3.4790 ± 0.3537 | 3.2254 ± 0.3697 |
|  | Hind Femur Length | 11.4104 ± 0.9326 | 11.2979 ± 0.5753 | 11.1566 ± 0.9110 |  | 9.6395 ± 0.9308 | 8.7983 ± 0.7365 |
|  | Harp Area | 10.3436 ± 1.4098 | 10.1377 ± 1.2779 | 9.8841 ± 1.0700 |  | 7.4163 ± 0.8187 | 7.0024 ± 0.7002 |
|  | Mirror Area | 3.8129 ± 0.5051 | 3.9778 ± 0.6587 | 4.0067 ± 0.6331 |  | 2.8401 ± 0.5294 | 2.5144 ± 0.4016 |
|  | Number of Teeth in a File | 120.71 ± 0.294 | 118.90 ± 10.079 | 115.07 ± 8.429 |  | 148.78 ± 10.737 | 142.15 ± 14.113 |
| female | Head Width | 4.8873 ± 0.4424 | 4.8310 ± 0.3825 | 4.8003 ± 0.4122 |  | 4.3750 ± 0.3605 | 4.0963 ± 0.5911 |
|  | Thorax Length | 4.3729 ± 0.3963 | 4.2819 ± 0.5142 | 4.1317 ± 0.4539 |  | 3.9248 ± 0.4012 | 3.5700 ± 0.4001 |
|  | Hind Femur Length | 12.6369 ± 0.9238 | 12.0688 ± 1.0144 | 11.8930 ± 0.9780 |  | 10.8527 ± 1.0846 | 9.8637 ± 0.7199 |
|  | Ovipositor Length | 13.2140 ± 1.3723 | 13.4274 ± 2.1882 | 13.5877 ± 1.7093 |  | 11.1991 ± 0.8811 | 10.8793 ± 1.1298 |

The values in a cell are mean ± standard deviation. See the Method for definitions of morphological characters. See Table 1 for sample sizes.
